# Web of microbes (WoM): a curated microbial exometabolomics database for linking chemistry and microbes

**DOI:** 10.1186/s12866-018-1256-y

**Published:** 2018-09-12

**Authors:** Suzanne M. Kosina, Annette M. Greiner, Rebecca K. Lau, Stefan Jenkins, Richard Baran, Benjamin P. Bowen, Trent R. Northen

**Affiliations:** 1 Environmental Genomics and Systems Biology, Lawrence Berkeley National Laboratory, M/S 100PFG100, 1 Cyclotron Road, Berkeley, CA 94720 USA; 2 Joint Genome Institute, Lawrence Berkeley National Laboratory, 2800 Mitchell Drive, Walnut Creek, CA 94598 USA; 3 National Energy Research Scientific Computing Center, Lawrence Berkeley National Laboratory, 1 Cyclotron Road, Berkeley, CA 94720 USA; 4 Current Address: UC San Diego Health Sciences, University of California San Diego, La Jolla, CA USA; 5 Current Address: Intrexon Corporation, 1750 Kraft Drive, Blacksburg, VA 24060 USA; 6 Current Address: Baran Bioscience, LLC, 2150 Allston Way Suite 400, Berkeley, CA 94704 USA

**Keywords:** Microbiome, Web of microbes, Exometabolomics, Mass spectrometry, Mass spectrometry based metabolomics, Metabolite exchange, Metabolite footprinting

## Abstract

**Background:**

As microbiome research becomes increasingly prevalent in the fields of human health, agriculture and biotechnology, there exists a need for a resource to better link organisms and environmental chemistries. Exometabolomics experiments now provide assertions of the metabolites present within specific environments and how the production and depletion of metabolites is linked to specific microbes. This information could be broadly useful, from comparing metabolites across environments, to predicting competition and exchange of metabolites between microbes, and to designing stable microbial consortia. Here, we introduce Web of Microbes (WoM; freely available at: http://webofmicrobes.org), the first exometabolomics data repository and visualization tool.

**Description:**

WoM provides manually curated, direct biochemical observations on the changes to metabolites in an environment after exposure to microorganisms. The web interface displays a number of key features: (1) the metabolites present in a control environment prior to inoculation or microbial activation, (2) heatmap-like displays showing metabolite increases or decreases resulting from microbial activities, (3) a metabolic web displaying the actions of multiple organisms on a specified metabolite pool, (4) metabolite interaction scores indicating an organism’s interaction level with its environment, potential for metabolite exchange with other organisms and potential for competition with other organisms, and (5) downloadable datasets for integration with other types of -omics datasets.

**Conclusion:**

We anticipate that Web of Microbes will be a useful tool for the greater research community by making available manually curated exometabolomics results that can be used to improve genome annotations and aid in the interpretation and construction of microbial communities.

**Electronic supplementary material:**

The online version of this article (10.1186/s12866-018-1256-y) contains supplementary material, which is available to authorized users.

## Background

Metabolomics research has been accelerated by advances in mass spectrometry techniques [1–4] that have allowed for the characterization of changes in complex pools of exogenous metabolites to link microbes to chemical transformations in their environments. These ‘metabolite footprinting’ or ‘exometabolomic’ experiments compare the extracellular metabolite composition with and without microbes to determine which metabolites have increased or decreased as a result of microbial transformation of the media [5, 6]; workflows have been described in detail [7, 8] and generally involve the collection, extraction and analysis of extracellular metabolites; these may be from laboratory cultures or field samples (Additional file 1: Figure S1).

**Fig. 1:**
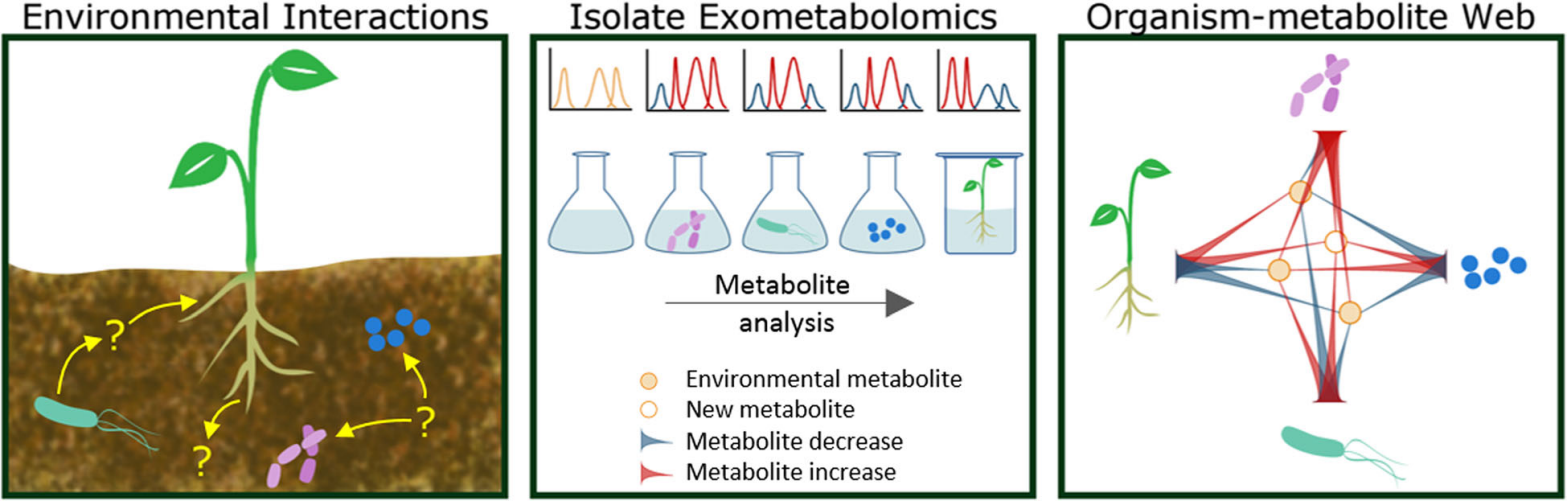
Isolate exometabolomics is used to elucidate microbe-environment interactions that shape the individual interactions within larger ecosystems. Unique, stable microbial communities mediate nutrient cycling within ecosystems; using microbiology and genomics approaches, the biotic and abiotic constituents, metabolic potential and interspecies interactions can often be identified. However, these interactions may be unique to a particular environment and the metabolites that mediate these interactions are difficult to identify within a community (left). By using exometabolomics analyses to examine the interactions between individual species and environments (ex. soil extract or plant root exudate) within a laboratory setting (middle), we are able to build a metabolite-organism interaction web demonstrating how each biotic component contributes to the community (right) enabling predictions for metabolite exchange in microbiomes, design of synthetic media, and testing of hypotheses using in lab consortia and environmental amendments

While current metabolomics databases typically contain information relating organisms, intracellular metabolites, chemical properties, metabolic pathways, spectral data, and occasionally other -omics type data (genomics, transcriptomics, proteomics) [9], to our knowledge, there is currently no data repository for exometabolomics studies. Exometabolomic based microbiology research is gaining widespread use for investigations in a variety of areas including rhizosphere interactions [10], overflow metabolism [11], optimization of biofuel feedstock production [12], chemical communication [13], metabolic footprinting of microbial communities [14], and microbial contamination detection [15]. Sequencing data generally falls short without functional studies when predicting interactions within an environment (Fig. 1). Exometabolomics is a powerful complement to sequencing approaches (Fig. 1) and has potential to help improve genome annotations [6, 16]. Thus, a publicly accessible exometabolomics repository would benefit and complement the fields of functional genomics and microbiome research.

**Fig. 2:**
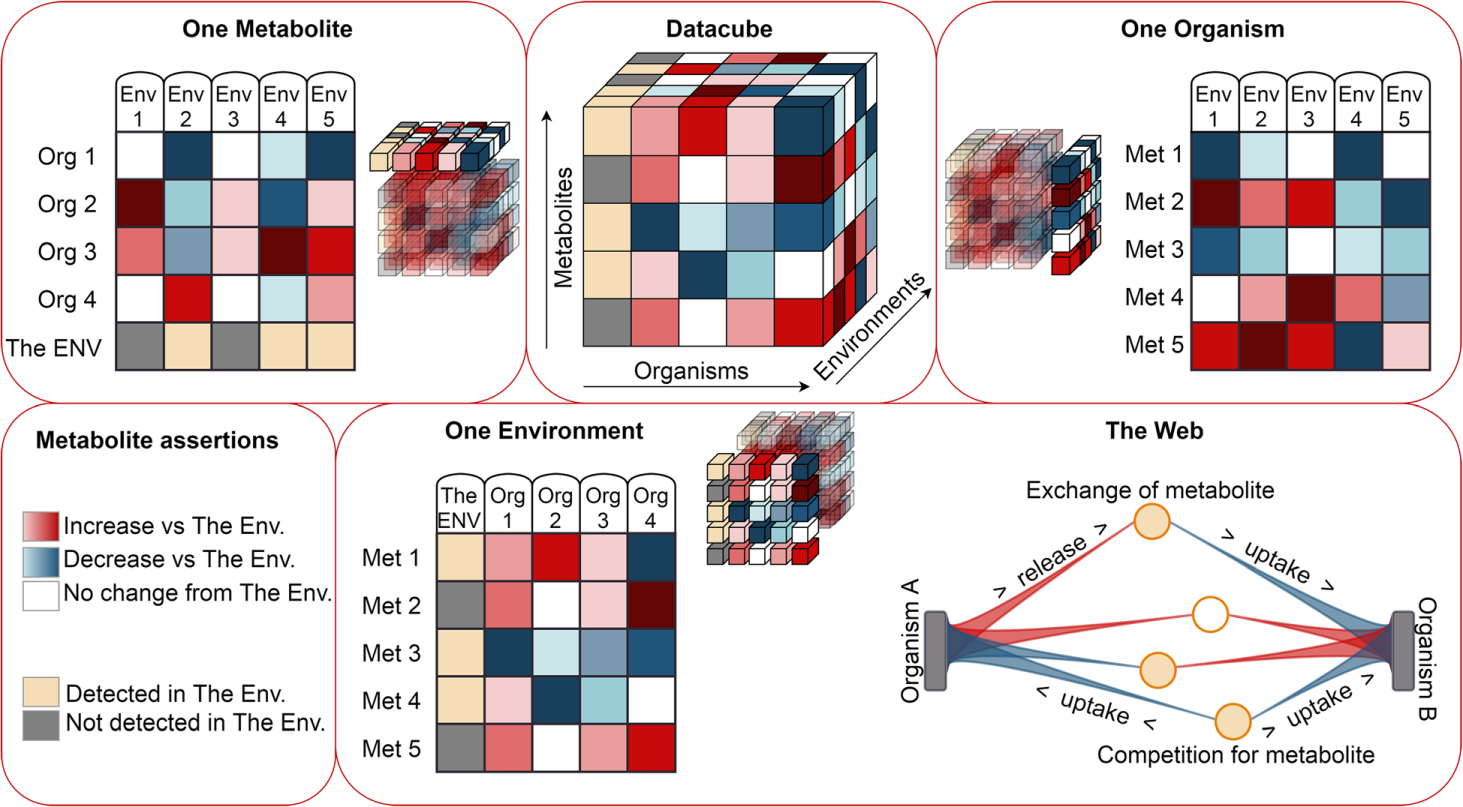
The Web of Microbes datacube and metabolic interaction web. Data on the WoM is sliced into two-dimensional views. For the control (“The Environment”), metabolites are “present” (tan) or “not detected” (gray). In the metabolically transformed environments (indicated by organism name), metabolites are coded as “decreased” (blue), “increased” (red) or “not changed” (white). Data constrained by selection of an environment can also be viewed in the form of a web. The size of each circle (metabolite) in the center of the web is relative to the number of organisms interacting with the metabolite. Filled circles indicate the metabolite was detected in the control exometabolite pool. Hollow circles indicate products not detected in the control environment. Connecting lines indicate metabolite increases (red) or decreases (blue) in the transformed metabolite pool

Here, we describe the Web of Microbes (WoM), freely accessible at http://webofmicrobes.org. This web-based data visualization tool and data repository for mass spectrometry based exometabolomics studies enables linking microbes, metabolites, and environments. WoM currently contains many carefully curated exometabolomics datasets from a single laboratory’s work. The web interface displays information from a three-dimensional dataset where a single exometabolomic observation is defined by a unique combination of metabolite, environment and organism data (Fig. 2). The measured observations indicate a qualitative assessment of the metabolite abundance relative to a control, thus indicating metabolite increase or decrease in the presence of the microorganisms; the measured observations for a control environment simply represent detection of the metabolites. In all samples, detection represents confirmed metabolite identification above the limits of detection for the analytical instrument that was used; identification is based the reporting standards as defined by the Metabolomics Standards Initiative (MSI) and typically includes at least two independent, orthogonal properties (for example, chromatography retention time and mass spectrometry fragmentation spectrum) compared with a pure reference standard [17]. Several examples are provided showing how data on the WoM can be sliced so that any two dimensions can be viewed in table format. Additionally, organism-metabolite data within a single environment can be viewed as a network (“The Web”) of interactions between metabolites and organisms.

## Construction and content

### Design

Web of Microbes (WoM) is hosted at the National Energy Research Scientific Computing Center (NERSC). The availability of high-end computing resources, together with NERSC’s robust science gateway infrastructure, should enable WoM to grow both in volume and features in a stable and reliable environment. WoM is a python application, built on the Django web development framework. It is served from a self-contained python environment on the NERSC global filesystem by an Apache web server. Visualizations are created with JavaScript, cascading style sheets (CSS) and the D3 JavaScript visualization library (http://d3js.org/). As the user interacts with the visualizations, asynchronous JavaScript and XML (AJAX) calls are made to the python backend to retrieve data from an SQLite3 (https://www.sqlite.org/) database. The calls are based on an application programming interface (API) approach known as Representational State Transfer (REST), forming a REST-based API. Content returned by the server does not depend on the state stored within the application. Taking this architectural approach should ease future integration with other bioinformatics tools, such as Kbase (https://kbase.us). Because the application leverages Django’s model–view–controller (MVC) structure, WoM also has good separation between front and back ends, making the data model and control logic easy to repurpose. While much of the application uses JavaScript, the need to make WoM embeddable in other systems made it important to separate the client-side data manipulation from the rendering and to take advantage of responsive design techniques, such as the use of Bootstrap’s fluid containers and CSS3. The network visualization on the “The Web” tab is a custom layout built with the D3 JavaScript library, while the heatmap-like table visualizations on the “One Metabolite/Environment/Organism” tabs take advantage of JQuery and JavaScript-manipulated HyperText Markup Language (HTML) tables with CSS styling. WoM runs on all modern web browsers.

### Datacube structure and user Interface

WoM has been designed to allow the user to obtain information relating three dimensions of data: (1) organisms, (2) metabolites and (3) environments in a number of different ways (Fig. 2). Data from these three dimensions can be uploaded to the Web of Microbes, where the user can constrain one of these dimensions to view the data as a two-dimensional table or web of assertions. Four different views are available by selecting the tabs labeled “The Web”, “One Environment”, “One Organism” and “One Metabolite”. “The Web” view displays data constrained by the selection of an environment (Fig. 2), while the other three tabs display data constrained by the dimension indicated by the selected tab’s label: “One Environment” (increase and decrease of multiple metabolites by 1 or more microbes within a single environment), “One Metabolite” (increase and decrease of a single compound by 1 or more organism in 1 or more environments) and “One Organism” (increase and decrease of multiple metabolites by a single organism in 1 or more environments). When constraining data by an environment (The Web, One Environment) or an organism (One Organism), the user selects the constraining dimension from a dropdown list on the upper part of the selection panel. When constraining data by a compound (One Metabolite), selections are made via a search box rather than a drop-down menu; note that only part of the name of a metabolite is required for searching. For example, ‘aden’ can be used to search for all compounds containing that combination of letters (adenine, adenosine, methyladenosine, etc). The unconstrained dimensions are represented in the table columns and rows. For each of the views, the values of the dimension in the column headings can be selectively added or removed from the table via check boxes on the lower part of the selection panel. Table cells and web visualizations follow a consistent color coding scheme – metabolite data for control environments are displayed as gray (not detected) or tan (detected) while data for biotically transformed environments are displayed as red (increased), blue (decreased), or white (no net increase/decrease). Checkered gray/white indicates the metabolite was not investigated during the data analysis. Terminology is further defined in Additional file 2.

### Database

Data sets are stored in an SQLite database (Additional file 1: Figure S2) which is available for download from the "About" tab on the WoM website. Free tools are available via the web for interacting with SQLite databases, such as the WoM database. The WoM database is relational, tying tables that describe individual environments, organisms, and metabolites together as defined observations of actions on metabolites. Observations are also tied to a projects table, in order to track data entry and ownership. Since SQLite data exists in a single file, we can readily share the data and relationships by allowing downloads of a simplified version of our database file. We have also built a data uploader that enables investigators to add data in comma-separated values (CSV) file format via a simple web interface. The small upload form triggers a python function that parses the data for multiple observations from a single input file. Data uploads and administration are performed on an internal version of the site so that new experimental results can be made available to staff before transitioning to the public site. The database will be maintained at Lawrence Berkeley National Laboratory by members of the Northen Metabolomics Group.

### Data preparation and upload

The data displayed on the WoM are the end results of exometabolomic workflows (Additional file 1: Figure S1). Metabolite analysis may be performed using any instrumentation/software that outputs relative metabolite abundances for each metabolite-organism-environment combination tested and analyzed. Using the relative abundance data, WoM data processing (Additional file 1: Figure S3) can be performed using R, python, spreadsheet formulas or other analysis tools. Briefly, metabolite components of the untransformed environment (control) are characterized as detected or not detected. The components of the transformed environment (spent) are first held to the same threshold for presence. If the compound is below the level of detection (not asserted as present) in both the control and spent media, no further assertions are determined. If the metabolite is asserted as present in either the control or spent media or present in both the control or spent, then control and sample are compared for statistically significant differences. The type of statistical and post hoc testing is dependent on the design of the experiment and are defined in the respective methods sections for each published dataset or in SI Additional file 1 for the use case “Exometabolomics of soil isolates in R2A”. A lack of significance is asserted as “no change”. For statistically significant differences, the log2 fold change (calculated as the log 2 of the measured value divided by the control value) between the spent and control media is used for table cell shading (and web connector colors). On the table views, darker shading of the red (metabolite increase) and blue (metabolite decrease) cells indicates a larger fold change. For cases in which the metabolite was detected in either the spent or control media, but not both, and the statistical test indicates significance, a max score of 5 is given for confidence of increase or decrease, since log2 fold change with respect to zero would result in an error. Observation data is uploaded via a tab-delimited text file containing, at a minimum, the following column headers: metabolites, organisms, environments and action assertions (increase, decrease, no change, detected and not detected). Additional headings/values, as available in the database structure (Additional file 1: Figure S2), may be included or manually entered via an admin console on the website.

### Sources of data

Currently, Web of Microbes contains curated data produced by the Northen Metabolomics Laboratory using liquid chromatography – mass spectrometry (LCMS) based techniques with the goal of illustrating use cases and making these data available to larger groups of scientists. At this time, we do not allow direct upload given the importance of careful data curation. Individuals interested in contributing data are encourage to contact the corresponding author. Any analytical method that can accurately identify and measure relative metabolite abundance in a control versus treatment group is acceptable; however the inclusion of evidence for accurate data curation is imperative. For now, level 1 identifications [17] are indicated using the name of the reference compound for which there was a match in the sample; putative identifications are indicated with the name in parentheses and where only limited structural information is available, as much information as available is used (ex. list of isomers, chemical class, molecular formula, or the peak’s retention time and m/z value). Currently, metabolite identification tables including metabolite, organism, environment and peak abundance were obtained from previously published works as indicated in Table 1 with the exception of the experiment titled: “Soil bacteria carbon utilization in complex culture medium” (methods and metabolite identifications are described in Additional file 1 and Additional file 3, respectively).

**Table 1:** Web of Microbes Data Examples

Field/Use-case	Example in WoM	WoM feature^a^
Ecosystem biology	Biocrust bacteria interactions with *Microcoleus vaginatus* metabolites [18]	The Web, One Metabolite
Metabolic responses to metabolite composition	Comparison of *Synechococcus* sp. in four types of growth media [8]	One Organism
Environmental microbiology	Soil bacteria carbon utilization in complex culture media (see Additional file 1: Appendix file methods)	One Environment, EUS, OCS-FME, OCS-FMC
Biotechnologies	Exometabolomics for design of synthetic mutualism systems [21]	OCS-FME
Native consortia	Biocrust porewater native microbial community incubations [18]	One Environment
Metabolic changes over time	Triculture of soil microbes in amino acid medium [19]	One Environment

^a^The Web, One Metabolite, One Organism and One Environment are all viewing features available on the Web of Microbes. The EUS, OCS-FME and OCS-FMC are compatibility scores available on the One Environment view. All features and WoM terminology are defined in Additional file 2: Appendix A

### Compatibility predictions within one environment

Compatibility scores are displayed in the “One Environment” table view and are based upon the assertion values in the control and transformed media. They are displayed in the column headings in relation to a user-selectable reference column which is highlighted in yellow. The reference column can be changed by clicking the desired column heading. At this time, three compatibility scores are available: an Environmental Uptake Score (EUS) and two Organismal Compatibility Scores (OCS). Selection of “The Environment” as a reference column will display EUS scores for each of the organisms and selection of one of the organisms as a reference column will display OCS scores for all non-reference organisms calculated with respect to the reference organism.

The EUS provides an approximation of compatibility between organisms and an available pool of metabolites. When “The Environment” is selected as a reference column, the EUS is calculated for each organism and represents the fraction of metabolites that an organism consumes (decrease) from the starting metabolite pool or environment (Eq. 1).

1 $$ EUS=\frac{number\kern0.17em of\kern0.17em metabolites\kern0.17em decreased\; by\; scored\kern0.17em organism}{number\kern0.17em of\kern0.17em metabolites\kern0.17em present\; on\; The\kern0.17em Environment} $$

OCSs are calculated between two organisms individually cultured in the same environment. Two different scores are used to assess the interorganismal compatibility: the FMC or fraction of metabolites under competition and the FME or fraction of metabolites for potential exchange. Potential for metabolite competition is indicated when two organisms decrease the same metabolite and represents the fraction of metabolites the scored organism decreases that are also decreased by the reference organism (Eq. 2).

2 $$ OCS- FMC=\frac{number\kern0.17em of\kern0.17em metabolites\kern0.17em decreased\; by\; both\kern0.17em organism}{number\kern0.17em of\kern0.17em metabolites\kern0.17em decreased\kern0.17em only\; by\; the\kern0.17em scored\kern0.17em organism} $$

When there is anti-correlated action (one organism decreases the metabolite and the other increases the metabolite), the organism that consumes or decreases the metabolite may be metabolically benefitting from the exchange; in these cases we represent this potential for exchange with the FME score (Eq. 3) which for the scored organism represents the fraction of consumed metabolites that are potentially provided for by the reference organism.

3 $$ OCS- FME=\frac{number\kern0.17em of\kern0.17em metabolites\kern0.17em decreased\; by\; the\kern0.17em scored\kern0.17em organism\kern0.17em and\kern0.17em increased\; by\; the\kern0.17em reference\kern0.17em organism}{number\kern0.17em of\kern0.17em metabolites\kern0.17em decreased\kern0.17em only\; by\; the\kern0.17em scored\kern0.17em organism} $$

A high degree of metabolic compatibility is inferred from a low degree of competition and high degree of potential for exchange. When the reference column is an organism, competing and compatible interactions between organisms are marked in table cells by symbols in the table cells (Additional file 1: Figure S7). It should be noted that the scores are a gross simplification used only for making predictions on compatibility and ignore the importance of the metabolite’s function (e.g. whether it is used in central carbon metabolism benefitting the recipient vs. a toxin that the recipient is degrading at an energetic cost).

## Utility and discussion

Use cases are presented to demonstrate the various features within the Web of Microbes and to highlight the value of this new repository in understanding metabolite exchange between representative organisms and their environments (Table 1). At this time, the data repository includes results from internal projects that were manually curated to ensure consistent and high quality metabolite identifications.

**Fig. 3:**
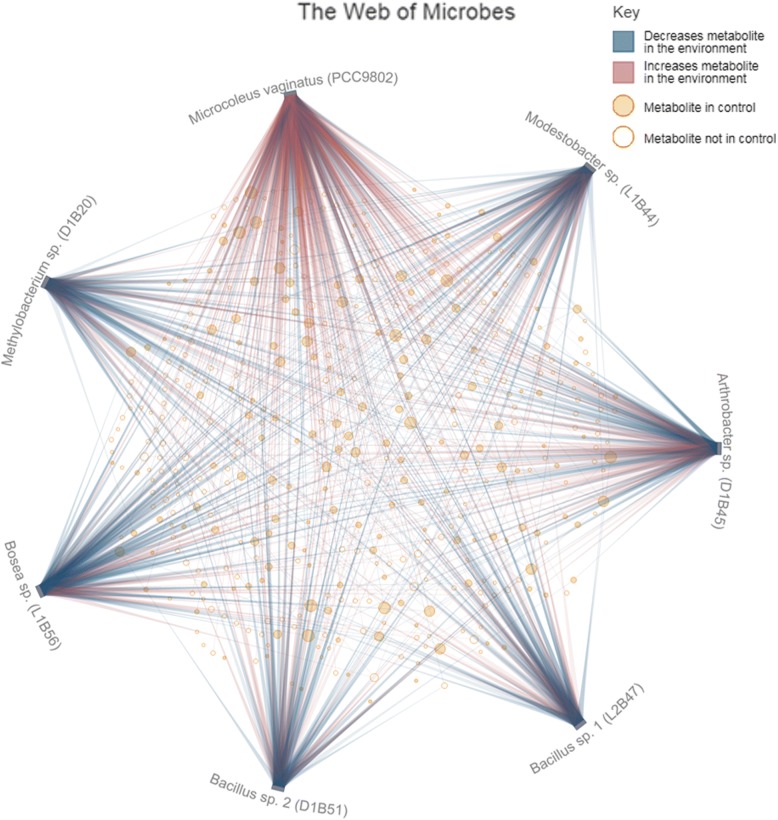
WoM The Web: Microcoleus vaginatus and six heterotrophic biocrust isolates in *M. vaginatus* extract. *M. Vaginatus* extract supplemented BG11 minimal medium was analyzed for metabolite composition before and after mono-culture of *M. vaginatus*, *Bosea* sp. Strain L1B56, *Methylobacterium* sp. Strain D1B20, *Modestobacter* sp. Strain L1B44, *Arthrobacter* sp. Strain D1B45, *Bacillus* sp. 1 Strain L2B47 and *Bacillus* sp. 2 Strain D1B51 [18]. The metabolite composition of the control medium is represented by the solid tan circles. Hollow circles are metabolites that were only identified after microbial transformation (indicating production/release by at least one of the organisms and not initially present in the control medium). Connecting lines indicate an increase (red) or decrease (blue) in the metabolite level in the spent medium compared to the control. Metabolite names are displayed using a toggle button on the website

The Web view provides an overview of metabolite and organism interactions within a single environment. In a recent experiment [18], BG11 (Blue-Green) minimal medium (a formulation optimized for cyanobacteria containing citrate, trace metals and essential nutrient salts) was supplemented with an extract of intracellular metabolites from *Microcoleus vaginatus* (PCC9802), a primary producer in biocrusts of the Colorado plateau. *M. vaginatus* and six heterotrophic isolates from Colorado plateau biocrust (phylotypes commonly associated with the primary producers) were mono-cultured in the *M. vaginatus* supplemented BG11. Using The Web view, it is evident that *M. vaginatus*, even in its own cellular extract, increases many compounds (Fig. 3). From this view, there is a visible disparity in the metabolic interactions when comparing *M. vaginatus* (many red /increase connecting lines) to the 6 heterotrophs (fewer red/increase, more blue/decrease). Interestingly, *M. vaginatus* also ‘consumes’ many of its own intracellular metabolites. While The Web is useful for rapid and qualitative assessment of metabolite interaction patterns, the table views provide a more detailed presentation of the data.

The three table view tabs each allow for the constraint of one dimension of data within the organism-metabolite-environment data model. For example, in the One Organism table view, selection *Synechococcus* sp. PCC7002 allows for the comparison of its metabolic actions in multiple starting metabolite pools [8]; in the A+ environment (no alanine available in control medium), alanine synthesis is stimulated whereas in A+ with *Synechococcus* sp. extract, alanine may be similarly produced or potentially, a byproduct of catabolic release from larger biopolymers present in that environment (Additional file 1: Figure S4). The One Metabolite view may be used to evaluate metabolic actions on a single compound across multiple different environments transformed by multiple different organisms; for example, glycine betaine, evaluated after incubation of biocrust isolates and native communities [18], was released by only one of the nine organisms and in only two of the three environments (Additional file 1: Figure S5). The One Environment view facilitates visualization of patterns on actions across multiple metabolites and organisms. For example, with ten soil isolates profiled in Reasoner’s 2A (R2A) broth medium, adenine and aspartic acid are decreased by all tested organisms, and similarly, creatinine and hydroxyproline are increased by most of the organisms tested while some metabolic interactions are unique to specific organisms: cytosine and alpha-aminoadipic acid are decreased by FW507-8R2A but increased by all other isolates tested (Additional file 1: Figure S6).

In addition to the three tables and the Web, a scoring system has been implemented for selecting potential microbial partners/competitors from the One Environment tab. Compatibility scores may be used for predicting both organism – environment and organism – organism interactions. Two isolates cultured in R2A medium, Pseudomonas sp. FW300-N2A2 and *Phenylobacterium* sp. GW123-8A04, were selected to demonstrate the use of the compatibility scoring system. The EUS scores indicate GW123-8A04 (EUS = 0.5) uses more of the existing resources than does FW300-N2A2 (EUS = 0.3). There are 25 metabolites that both organisms decreased, indicating possible competition for these resources; for GW123-8A04 these 25 are only 50% of the total it decreases (FMC = 0.5), whereas for FW300-N2A2, these 25 are 96% of the resources it uses (FMC = 0.96). When evaluating resources that may be exchanged between the two, there are no metabolites that are both increased by GW123-8A04 and decreased by FW300-N2A2 (FME = 0), thus FW300-N2A2 would not benefit from this interaction; however, FW300-N2A2 increases 8 metabolites that GW123-8A04 decreases (FME = 0.16). Combined, the scores indicate that GW123-8A04 is more likely to benefit from the interaction and potentially outcompete FW300-N2A2 (Additional file 1: Figure S7).

The WoM can be used to view data with additional factors to consider such as time points, synthetic consortia, native microbiomes and theoretical actions. In a recent study, time course analysis of three isolates mono-cultured in a defined amino acid medium was used to predict a model for amino acid consumption by a consortium containing all three isolates; the experimental tri-culture consumption data fit the model [19]. The tri-culture time-point results have been uploaded to the WoM each as unique ‘organisms’ to be compared in the One Environment or Web views showing the consumption patterns over time (Additional file 1: Figure S8). Native consortia are be displayed in a similar manner. Exometabolites were collected and analyzed after resuscitating a dormant biocrust [18]. Different pools of metabolites were decreased and increased at two time-points after activation with overall use at ~ 30% for each (Additional file 1: Figure S9), likely due to successional changes and ‘activation’ of different species over time which are known to occur in biocrust after ‘rainfall’ type events [20]. In a study illustrating the design of a synthetic mutualistic co-culture, wild-types of *E. coli* and *Z. mobilis* were analyzed in minimal medium (glucose as sole carbon source) for production of specific metabolites required to rescue auxotrophs of the opposite species [21]. The wild-type experimental data was used to show predicted metabolite exchange between auxotrophs of each strain (Additional file 1: Figure S10).

Additional uses of WoM include facilitation of experimental design, improved visualization of functional genomics annotations, improved interpretations of catabolite repression data. For example, in a previous study, 4141 transposon mutants of *Shewanella oneidensis* MR-1 with mutations in 3174 genes were cultured in a minimal medium (vitamins, metals, basal salts and lactate) supplemented with citrulline; this approach identified genes important for citrulline utilization: SO3749/SO0277, SO1141, SO1142 (a non-homologous functional analogue of argE) and SO1043/SO1044 (subunits of an ATP-binding cassette (ABC) transporter) [14]. This type of data could be compared in subsets within WoM on the One Environment tab. One Organism may be useful for interpretation of catabolite repression data when using defined media; this would allow easy comparison of metabolite preferences. Finally, a major but perhaps inconspicuous capability is the comparison of the compositions of multiple untransformed controls from the One Organism tab by constraining the data to “The Environment” (Additional file 1: Figure S11). This could be of use for identification of an environment with specific/desirable metabolites or for designing a synthetic medium based upon a representative environmental sample.

Finally, a discussion of a few caveats with the prediction scores, color scales and action assertions within the WoM is required to prevent misinterpretation of visualizations and data. Scores do not account for production of antibiotics, growth or other regulatory factors by one/both organism(s) or catabolite repression that may change the metabolic functions if cultured together. The red and blue color scales indicate certainty of qualitative difference from control, not quantitative abundance. Thus, care should be taken to only make comparisons between action types (increase/decrease) not abundance. Further, an action assertion (increase/decrease/no change) is the result of the net turnover for a given metabolite. Some metabolites will both be consumed and produced by the organism, possibly at different times during growth; this is especially important when considering “no change”, which could reflect a complete lack of interaction between the organism and the metabolite or may indicate equal rates of consumption and production. For co-eluting isomers that are not resolved via the analysis methods used (e.g. disaccharides in some LCMS methods), action assertions should be considered with caution, given that the individual actions on each of the isomers may differ. And lastly, an increase in a metabolite does not always indicate de novo synthesis. For example, FW300-N2E3, a predicted auxotroph for lysine, phenylalanine and tyrosine [22, 23], increases the amount of lysine and tyrosine in R2A (Additional file 1: Figure S6), possibly due to hydrolysis of peptides from the media.

## Conclusions

The Web of Microbes provides a valuable resource capturing exometabolomics data for asserting microbe-metabolite-environment interactions. This online repository and data visualization tool enables slicing an exometabolomics datacube into two dimensional views including “The Web”, “One Environment”, “One Organism” and “One Metabolite”. The Environmental and Organismal compatibility scores within the “One Environment” view allow users to make predictions of microbial compatibility within an environment. The data in the repository contains a variety of use cases, described here to exhibit inclusion of isolate and consortia data, native and synthetic communities, defined and natural environments, and data entry as single or multiple time points. A major next step in building the repository will be incorporation of additional in-house and external labs’ data. To accomplish this effectively, numerous challenges that must be addressed in order to control for experimental design considerations, data analysis quality, and use of common vocabulary. These goals will be met by gradually expanding the access and import capabilities, beginning with integration of existing programs at the Joint Genome Institute and Lawrence Berkeley National Laboratory where the resource is maintained (ex. metabolomics user program at Joint Genome Institute). In this manner, many issues relating to new types of data and quality of data collection/analysis may be addressed appropriately before more advanced access options are made available. Prior to adding any external data, a number of improvements to the database are required. For example, metabolite identification details such as Digital Object Identifier (DOI) or description of metabolite analysis methods, MSI identification level, etc. will be required on an assertion value basis when combining results from multiple studies within one slice of the datacube. Further, additional required fields will be necessary for describing experimental parameters (ex. time-points, incubation temperatures, source of metabolites, sources of organisms, etc.) to prevent overlaps in naming of environments/organisms. With the current and future versions, we anticipate that Web of Microbes will be useful to a wide variety of emerging fields, such as understanding the metabolites exchanged in syntrophic interactions [24], development of microbe-based carbon sequestration methods [25], engineered consortia for enhanced drug production/biofuels/bioremediation [26], and geomicrobiological applications for producing soil on extraterrestrial surfaces [27]. Future efforts will focus on expansion of the database, especially with collaborator and external user data as well as additional types of metabolomics analyses.

## Additional files

Additional file 1: Experimental considerations for exometabolomics. Metabolite identification considerations. Methods for Use Case: Exometabolomics of soil isolates in R2A. **Figure S1.** Exometabolomics workflows for Web of Microbes. **Figure S2.** Web of Microbes SQL Relational Database. **Figure S3.** Web of Microbes data processing: Metabolite assertions for control and spent metabolite pools. **Figure S4.** WoM One Organism view: The metabolic actions of Synechococcus sp. PCC7002 on four pools of metabolites. **Figure S5.** WoM One Metabolite view: The metabolic actions of multiple organisms in multiple environments on glycine betaine. **Figure S6.** WoM One Environment view: The metabolic actions of ten soil isolates in R2A medium. **Figure S7.** WoM Compatibility Scores: Predicted interactions of two soil isolates in R2A. **Figure S8.** WoM One Environment view: visualization of time-course and consortia data. **Figure S9.** WoM One Environment view: native microbial communities. **Figure S10.** WoM One Environment, OCS-FME score: Predicted metabolite exchange potential between auxotrophs. **Figure S11.** WoM One Organism view: Comparison of metabolite compositions of untransformed controls. (PDF 1599 kb)

Additional file 2: Appendix A. Web of Microbes Terminology. (PDF 414 kb)

Additional file 3: **Table S1.** microbial isolates, **Table S2.** LCMS parameters, **Table S3.** metabolite identifications, **Table S4.** ppm windows for experiments described in Additional file 1: Methods for Use Case: Exometabolomics of soil isolates in R2A. (XLSX 659 kb)

## Abbreviations

ABC transporter: ATP-binding cassette transporter
AJAX: asynchronous JavaScript and XML
API: application programming interface
BG11 Medium: Blue-Green Medium
CSS: Cascading Style Sheets
CSV file: comma-separated values file
DOI: Digital Object Identifier
EUS: Environmental Uptake Score
HTML: HyperText Markup Language
LCMS: liquid chromatography – mass spectrometry
MSI: Metabolomics Standards Initiative
MVC: model–view–controller
NERSC: National Energy Research Scientific Computing Center
OCS-FMC: Organismal Compatibility Score for the Fraction of Metabolites under Competition
OCS-FME: Organismal Compatibility Score for the Fraction of Metabolites for potential Exchange
R2A: Reasoner’s 2A
REST: Representational State Transfer
WoM: Web of Microbes
