# Supplementary material for: Web of microbes (WoM): a curated microbial exometabolomics database for linking chemistry and microbes
Source: BMC Microbiol. 2018 Sep 12;18:115. doi: 10.1186/s12866-018-1256-y (PMC6134592; doi:10.1186/s12866-018-1256-y)
Supplement: Supplementary file 1 — Experimental considerations for exometabolomics. Metabolite identification considerations. Methods for Use Case: Exometabolomics of soil isolates in R2A. Figure S1. Exometabolomics workflows for Web of Microbes. Figure S2. Web of Microbes SQL Relational Database. Figure S3. Web of Microbes data processing: Metabolite assertions for control and spent metabolite pools. Figure S4. WoM One Organism view: The metabolic actions of Synechococcus sp. PCC7002 on four pools of metabolites. Figure S5. WoM One Metabolite view: The metabolic actions of multiple organisms in multiple environments on glycine betaine. Figure S6. WoM One Environment view: The metabolic actions of ten soil isolates in R2A medium. Figure S7. WoM Compatibility Scores: Predicted interactions of two soil isolates in R2A. Figure S8. WoM One Environment view: visualization of time-course and consortia data. Figure S9. WoM One Environment view: native microbial communities. Figure S10. WoM One Environment, OCS-FME score: Predicted metabolite exchange potential between auxotrophs. Figure S11. WoM One Organism view: Comparison of metabolite compositions of untransformed controls. (PDF 1599 kb) [file 12866_2018_1256_MOESM1_ESM.pdf]

Web of Microbes (WoM): a curated microbial exometabolomics database for linking chemistry and microbes

Suzanne M. Kosina, Annette M. Greiner, Rebecca K. Lau, Stefan Jenkins, Richard Baran, Benjamin P. Bowen, and Trent R. Northen

Supplemental Data

Experimental considerations for exometabolomics........1
Metabolite identification considerations........1
Methods for Use Case: Exometabolomics of soil isolates in R2A........1
Figure S1. Exometabolomics workflows for Web of Microbes........2
Figure S2. Web of Microbes SQL Relational Database.........3
Figure S3. Web of Microbes data processing: Metabolite assertions for control and spent metabolite pools........4
Figure S4. WoM One Organism view: The metabolic actions of Synechococcus sp. PCC7002 on four pools of metabolites........5
Figure S5. WoM One Metabolite view: The metabolic actions of multiple organisms in multiple environments on glycine betaine........5
Figure S6. WoM One Environment view: The metabolic actions of ten soil isolates in R2A medium........6
Figure S7. WoM Compatibility Scores: Predicted interactions of two soil isolates in R2A.........7
Figure S8. WoM One Environment view: visualization of time-course and consortia data........8
Figure S9. WoM One Environment view: native microbial communities........8
Figure S10. WoM One Environment, OCS-FME score: Predicted metabolite exchange potential between auxotrophs........9
Figure S11. WoM One Organism view: Comparison of metabolite compositions of untransformed controls........10
References........11

Experimental considerations for exometabolomics: Comparison of samples in the Web of Microbes, relies on inclusion of a control treatment "The Environment" to compare with metabolically transformed environments, the organisms. Of note, metabolic transformation could also include the use of enzymes or other in vitro systems. Our focus is on exometabolomics thus workflows are described in the context of metabolite transformations by metabolically active organisms (Figure S1). This requires that the environment be treated under the same abiotic conditions as those used for the transformed environment. For example, in an experiment inoculating a broth medium with microorganisms, an un-inoculated control of sterile medium incubated under the same temperature/light/humidity/time should be included to account for abiotic transformations that could confound results (e.g. photodegradation of riboflavin). For some nonsterile environmental samples such as dormant biocrust that would be activated by wetting/warming/light, time zero would act as the control environment since sterilizing would release intracellular metabolites.

Metabolite identification considerations: Accurate identification techniques are essential for accurate downstream data preparation for upload to WoM; use of standard references, spectral databases, adduct analysis, fragmentation predictions and isotope abundances are some of the many tools to consider using when making identifications. In the WoM, in cases of putative metabolite identification, common metabolite name is used, e.g. adenosine. In other cases, as much identifying info as possible is used (e.g. C6H12O6). The identification criteria for use cases are described in published works or in the methods section below.

Methods for Use Case: Exometabolomics of soil isolates in R2A: Isolates were gifted from Romy Chakraborty (Lawrence Berkeley National Laboratory). Briefly, ground water from the Oak Ridge National Laboratory Field Research Center[1] (Oak Ridge, TN) wells FW300, FW507, FW104, GW101, GW123, GW456 and GW704 was streaked out on to solid media and incubated; isolates were collected from single colonies, stored in glycerol and identified by 16S sequencing or whole genome sequencing as indicated in Additional file 3: Table S1. For exometabolomics experiments isolates were resuscitated on R2A agar (Difco) solid medium at 30^oC, single colonies were used to inoculate R2A broth (Difco) liquid medium at 30^oC. After 24 hours, cells were washed by repeated centrifugation (3000 x g) and resuspension with fresh R2A and then adjusted to 0.02 OD at 600nm; these and control (uninoculated) samples were incubated in triplicate 3.5mL cultures in 5mL deep well pyramid bottom 48 well plates (Axygen). Spent media were collected at mid-log phase as determined by prior isolate growth curves. Cultures were centrifuged at 3000 x g for 3 minutes, 500uL of supernatants were filtered (0.2 um, GHP, PALL) to remove cells/debris, filtrates were dried by lyophilization, metabolites were reconstituted with 125 uL of methanol. Internal and external standards were used for quality control purposes. Extracts were sonicated and vortexed to expedite metabolite solubilization. Salts and other precipitates were pelleted by centrifugation (3000 x g, 5 min); supernatant was filtered (0.2um, PVDF, PALL) to remove residual particulates and filtrates were analyzed by LCMS. Extracts were separated using a hydrophilic interaction liquid chromatography (HILIC) column (zic-pHILIC column, 150 mm × 2.1 mm, 3.5 µm 100 Å, Merck SeQuant, Darmstadt, Germany) on an Agilent 1290 series UHPLC (Agilent Technologies, Santa Clara, California, USA) for analysis using a quadrupole time-of-flight mass spectrometer (Agilent 6550 Q-TOF-MS) with data acquired independently in positive and negative polarities with MS1 and automated MS/MS spectra

collection. Chromatography and mass spectrometry parameters are indicated in Additional file 3: Table S2. Using a python based metabolite atlas analysis workflow[2, 3] (code available for download at: https://github.com/biorack/metatlas), metabolite identifications were based on measured m/z ratio (at peak apex +/- 25 ppm) and retention times (+/- 75 seconds) vs. a retention time corrected atlas of authentic reference metabolites (metabolite annotations are provided in Additional file 3: Table S3). In SI Table S3 (Additional File 3), for any given metabolite-polarity pair, at least one mz_measured value was <8 ppm. Also, many were detected in both polarities, for example, adenine's mz_ppm range was between 2.79 and 24.87 in neg mode, but between 0.98 and 7.75 in pos mode (the mz_ppm ranges are listed in table S4). The polarity with the highest average intensity for treatment groups was used for the area measurements. Retention times for our in house reference standards library were collected on the same instrument, using the same methods. A subset of reference standards (external controls) was analyzed at the same time as the samples and used to generate the theoretical retention times via linear regression (this was done to account for slight shifts in retention over time due to changes in tubing length, different batches of mobile phase, different lot # of column from manufacturer, etc.). Raw data is available at: https://genome.jgi.doe.gov/portal/ENIBEMetabolites_FD. For statistical comparison of treatment versus controls, a two tailed T test was used ($p<0.05$). Identification tables are processed for WoM as described in the main text and Figure S3.

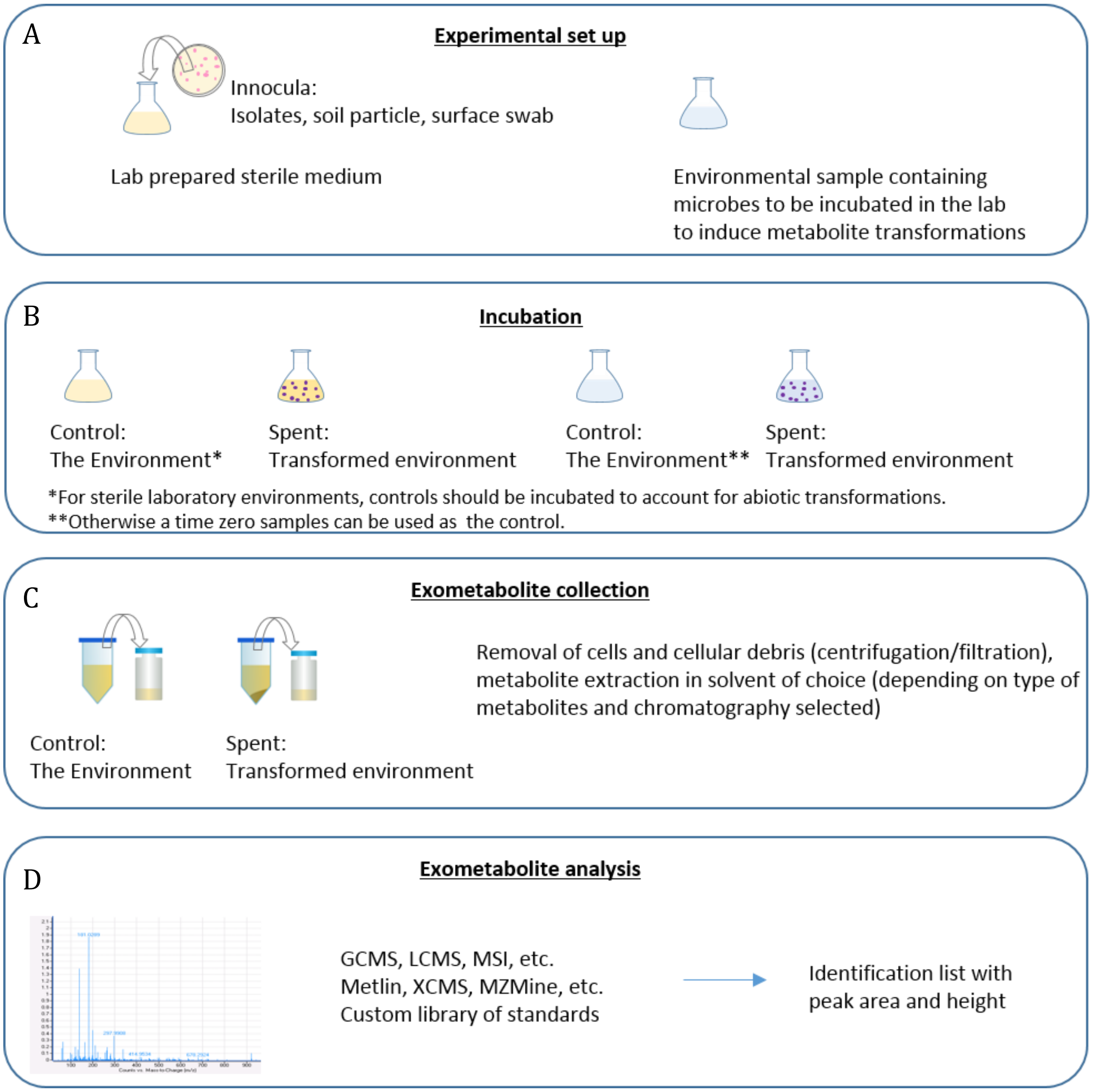

Figure S1. Exometabolomics workflows for Web of Microbes. (A) Environments (commercial growth medium, soil extract, cell lysates, etc.) and Organisms (isolates, native communities or synthetic consortia) are selected for evaluation. (B) Environments treated to investigate transformation of the metabolite pools, typically through inoculation with organisms. (C) After the environment has been transformed (either at multiple timepoints or a single end point), the exometabolites are collected by removal of the cellular components from the media. (D) The metabolite compositions of the control (untransformed environment) and spent (transformed environment) media are analyzed using mass spectrometry.

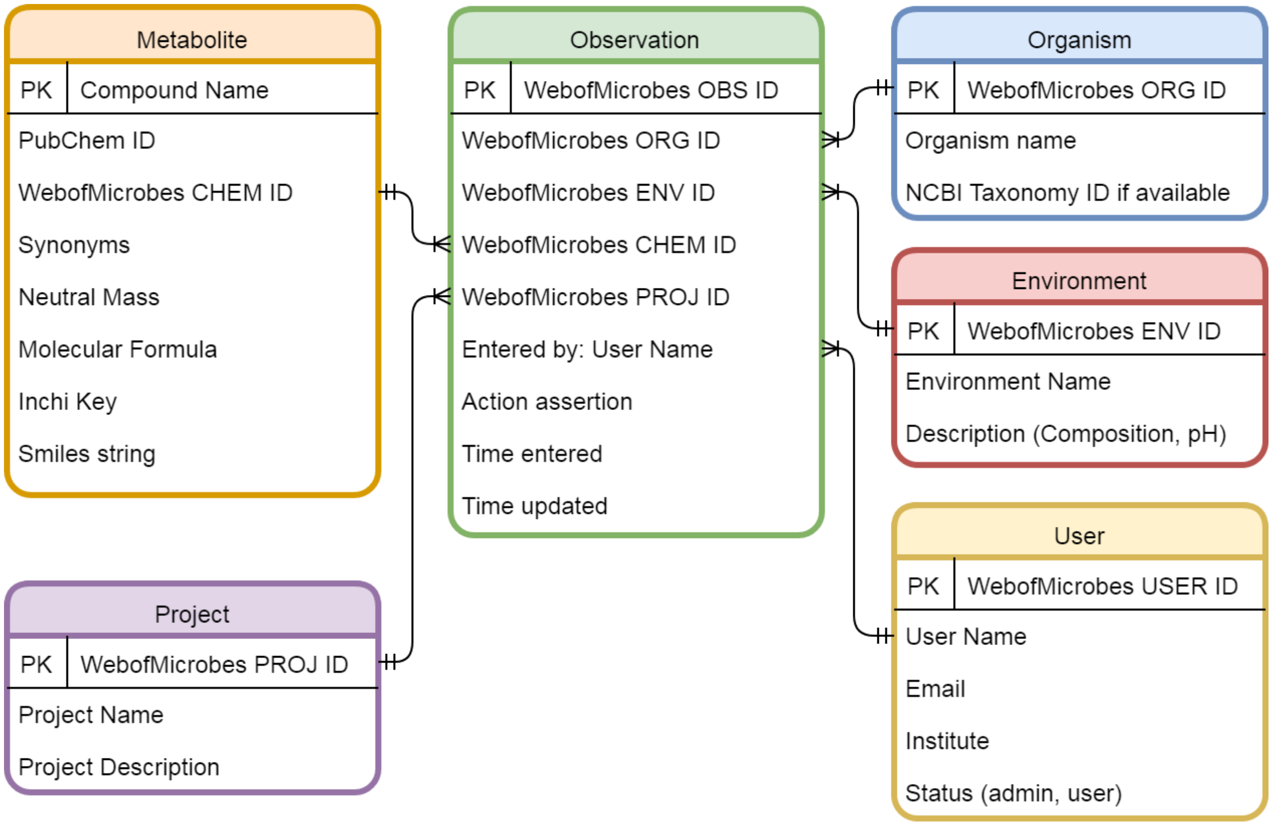

Figure S2. Web of Microbes SQL Relational Database. Simplified entity relationship diagram of the Web of Microbes database. Six application-specific major entities are stored. The attributes for the User are entered during account setup (at this time restricted to internal use only). All other attributes are entered during upload of Observation data and can optionally be edited via an administrator interface. In all cases, each Observation is tied to one and only one primary key for each of the other entities.

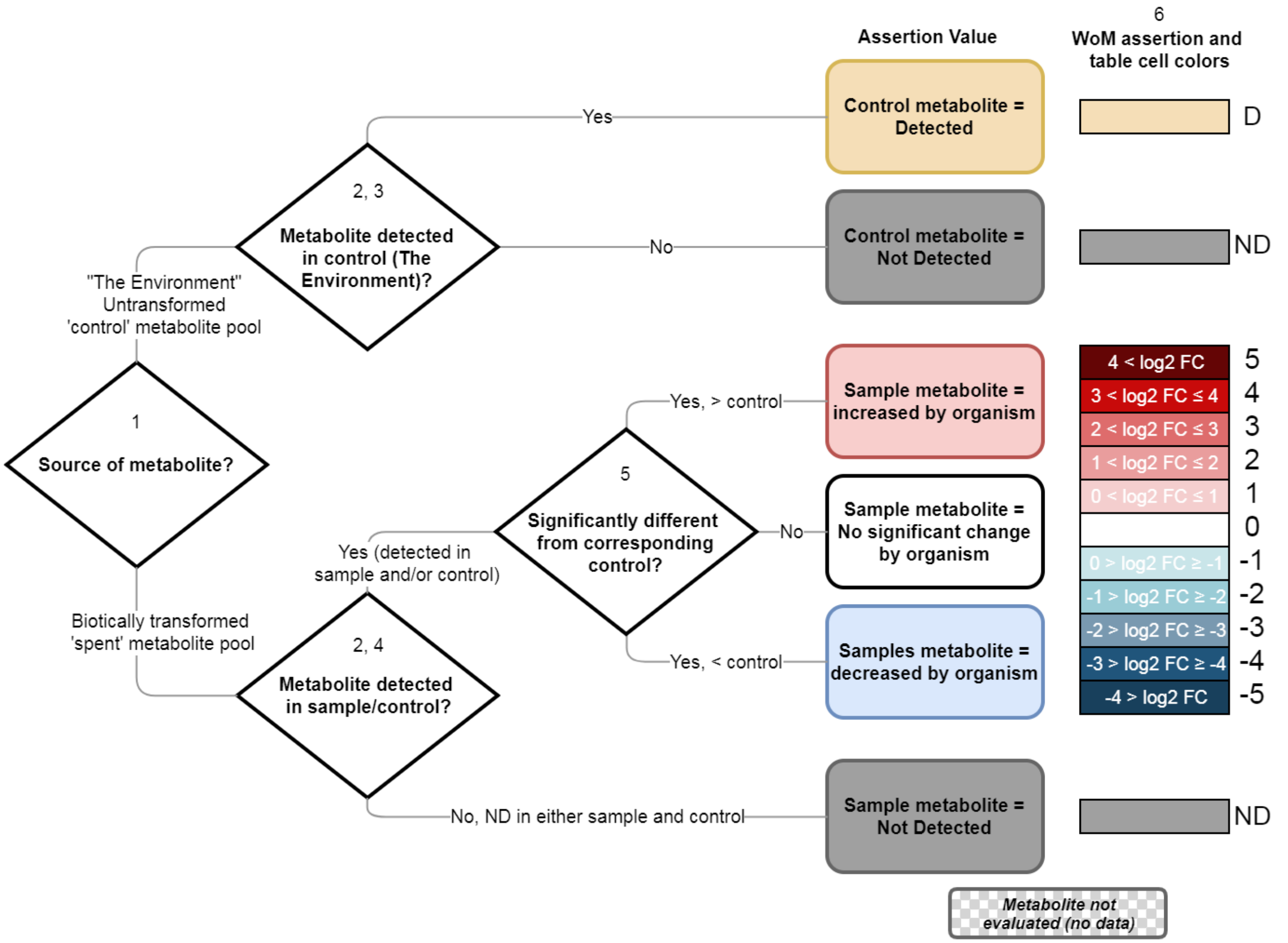

Figure S3. Web of Microbes data processing: Metabolite assertions for control and spent metabolite pools. After interpretation and analysis of exometabolomics results, the relative abundance data for each metabolite is used to determine assertions of metabolite detection (control) and actions (spent) through the following steps: (1) a sample is designated as a control ("The Environment") or treatment type (transformed/spent metabolite pool); (2) All samples are evaluated for detectable levels of the identified metabolite; limits of detection are determined experimentally for each experiment; (3) Control sample metabolites are asserted as 'detected' (tan) or 'not detected' (gray); (4) Treatment sample metabolites not detected in both the control or spent sample are asserted as 'not detected' (gray); (5) For metabolites detected in either or both of the control and spent samples, a statistical comparison determines actions for treatment samples which are asserted as 'increased' (red), 'decreased' (blue), 'not changed' (white); Metabolite actions of 'increase' or 'decrease' are shaded by log2 fold change cutoff values; (6) the website tables use the coloring and shading scheme as indicated.

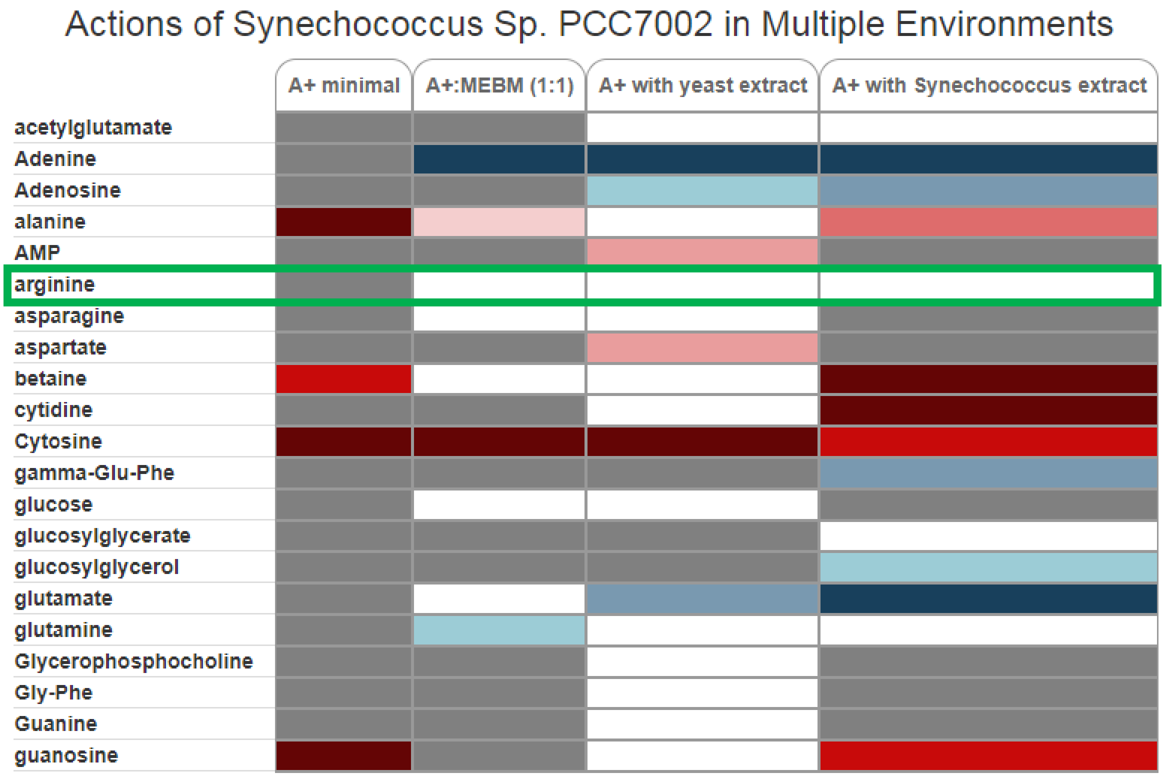

Figure S4. WoM One Organism view: The metabolic actions of Synechococcus sp. PCC7002 on four pools of metabolites. Four types of growth medium were analyzed before (control) and after (spent) culture of *Synechococcus* sp. PCC7002 [4]. The media types included: A+ (a buffered medium with trace components to support algal cultures), A+ with Yeast Extract (a rich medium providing common primary metabolites), A+ with *Synechococcus* extract (A+ supplemented with the intracellular extract of *Synechococcus* sp. PCC7002) and A+ with MEBM (A+ supplemented with a basal medium designed to support mammary epithelial growth). Note: only the first 24 metabolites in the table are displayed here. Cell colors are as described in Fig. S3.

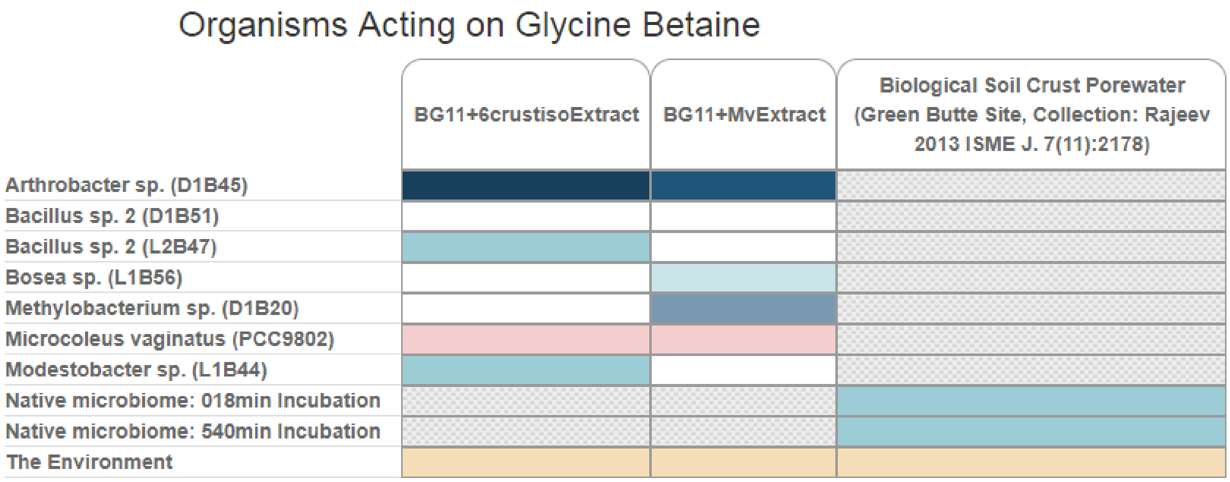

Figure S5. WoM One Metabolite view: The metabolic actions of multiple organisms in multiple environments on glycine betaine. In a previous experiment[5], BG11 minimal medium was supplemented with intracellular extracts from six isolates from the Colorado plateau biocrust or *Microcoleus vaginatus* PC 9802. Each individual organism was then cultured in the supplemented medium. Additionally, biocrust was transformed by native microbial communities by wetting, incubating for 18 or 540 minutes (time zero was used as the untransformed control) and collecting the porewater. While betaine is present in all starting environments (tan cells in The Environment row), it is only decreased/consumed by the heterotrophs and native

microbes (blue cells) while increased/released by the primary producer *M. vaginatus* (red cells). Cell colors are as described in Fig. S3.

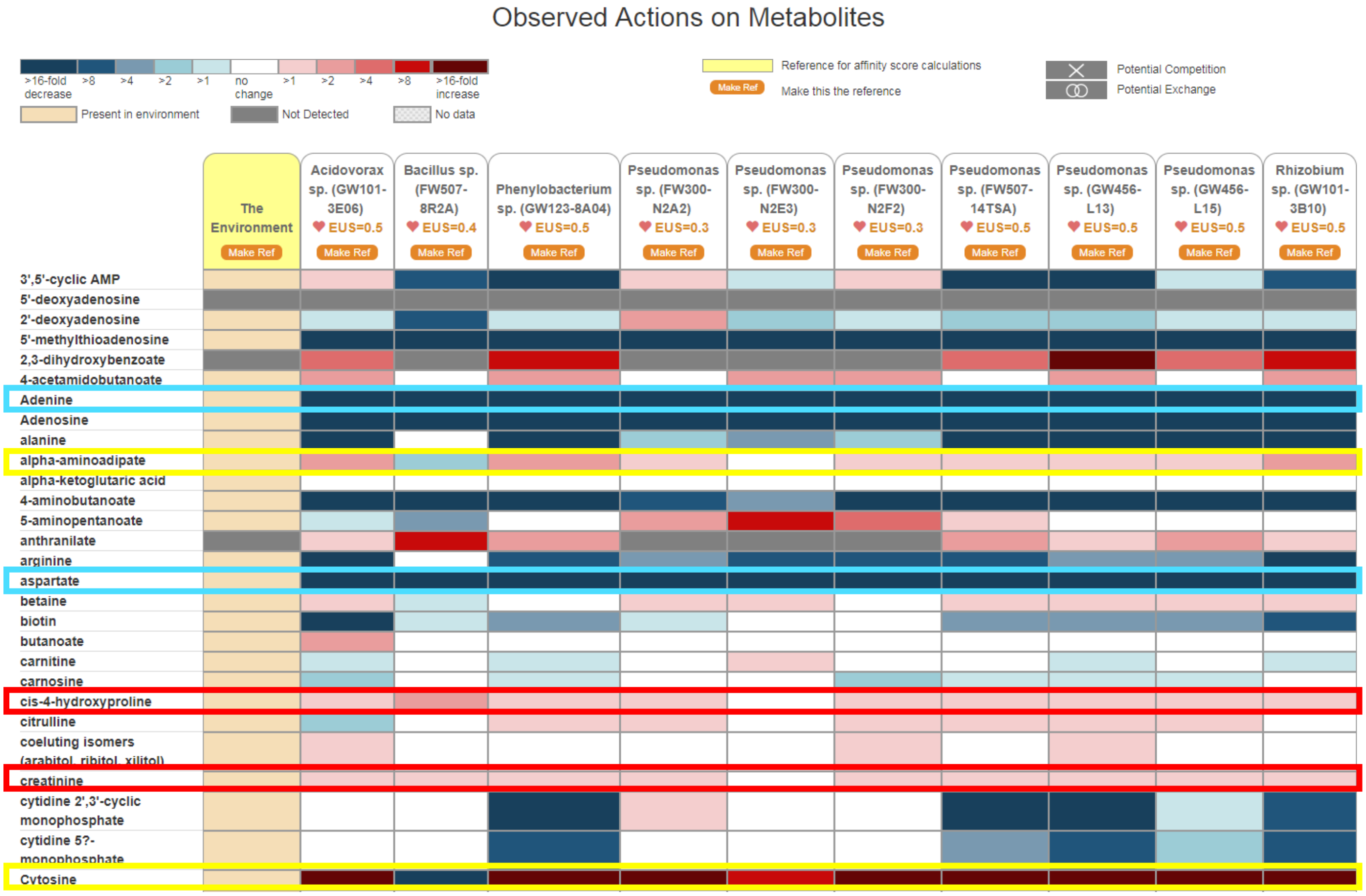

Figure S6. WoM One Environment view: The metabolic actions of ten soil isolates in R2A medium. R2A medium was analyzed before (control) and after (spent) transformation by each of ten strains isolated from the Oak Ridge, TN Field Research Center. Some metabolites show consistent utilization patterns across all organisms: adenine and aspartate are decreased by all (blue box), creatinine and cis-4-hydroxyproline acid are increased by most (red box); others have unique interactions only with certain organisms: cytosine and alpha-aminoadipic are decreased by one organism but increased by most of the others (yellow box). Note: Only the first 31 metabolites from the table are displayed here.

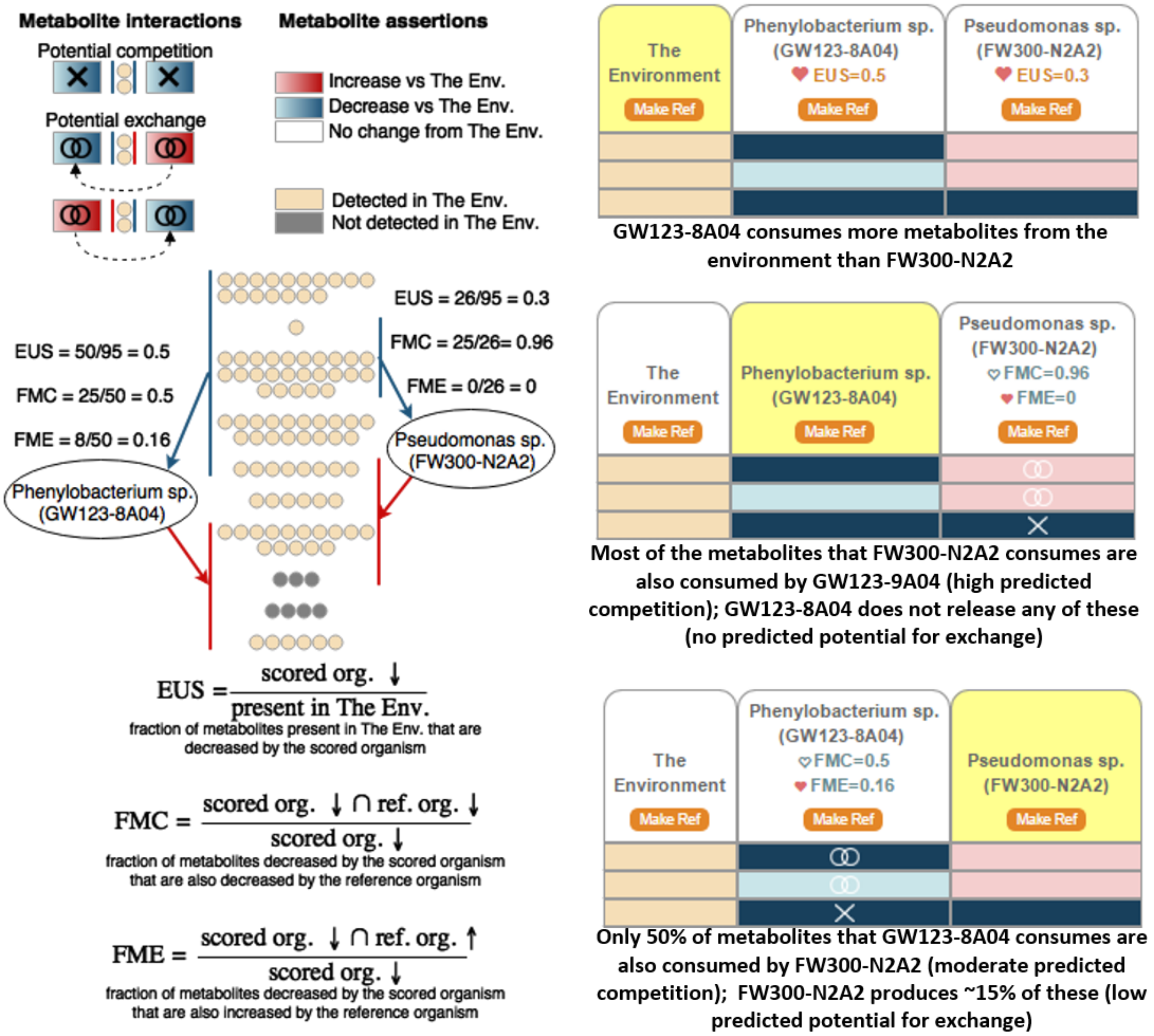

Figure S7. WoM Compatibility Scores: Predicted interactions of two soil isolates in R2A. Three scores are displayed on the One Environment tab: the Environmental Uptake Score (EUS), the Fraction of Metabolites under Competition (FMC) and the Fraction of Metabolites for potential Exchange (FME). An overview of the entire metabolite table (left) summarizes the calculations of these scores for two isolates, *Phenylobacterium* sp. (GW123-8A04) and *Pseudomonas* sp. (FW300-N2A2), in the environment of R2A liquid medium. Selection of "The Environment" (untransformed control) as the reference column displays the EUS for each of the organisms (upper right). Selecting one of the organisms as the reference column displays scores for all other organisms indicating potential organism-organism interactions. For example, with GW123-8A04 selected as the reference organism, the FMC and FME scores are displayed for FW300-N2A2 (right middle) and with FW300-N2A2 selected as the reference organism, the FMS and FME scores are displayed for GW123-8A04 (right lower). Additionally, the table cells are marked with interaction symbols: "×" indicates potential competition where both the scored and reference organisms decrease the metabolite (blue cell) and "⚭" indicates potential exchange where there are opposing actions (increase/decrease) between the scored and reference organisms such that one may produce a metabolite consumed by the other.

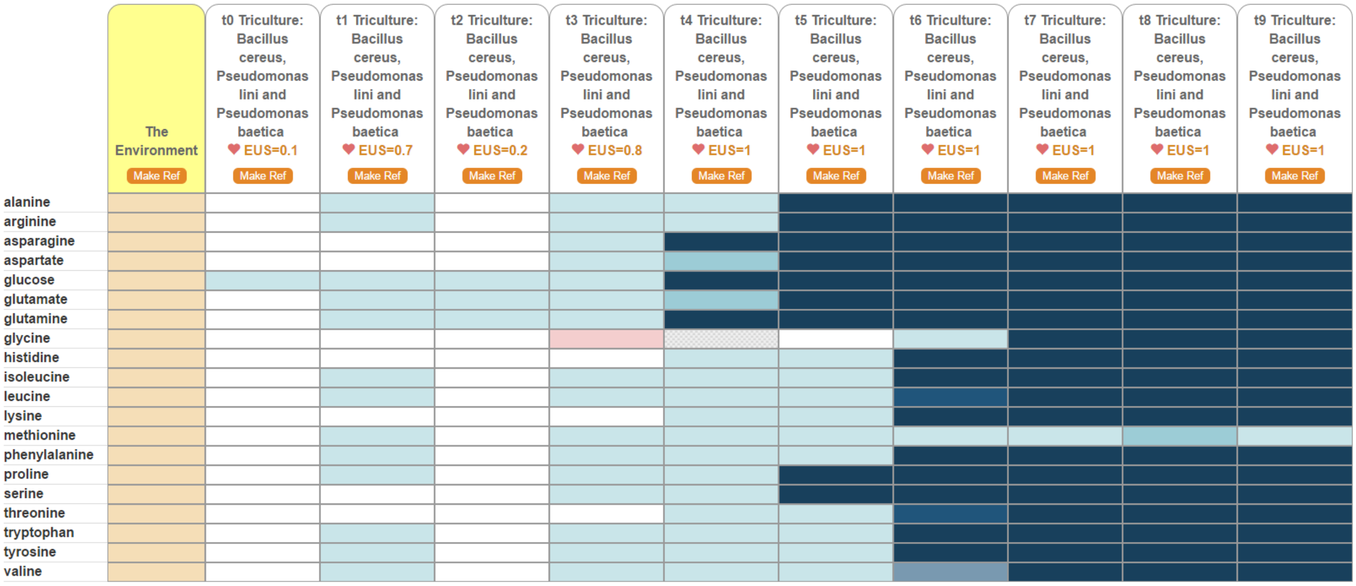

Figure S8. WoM One Environment view: visualization of time-course and consortia data. To demonstrate the incorporation of consortia and time course data, a defined minimal medium (glucose, minerals and vitamins) supplemented with amino acids was analyzed before (control) and after (spent) transformation by a triculture of *Bacillus cereus*, *Pseudomonas lini* and *Pseudomonas baetica*, three isolates from soil at the Oak Ridge Field Research Center[6]. With no significant abiotic changes over time in each time-point's uninoculated incubated control, the preinoculated control medium was used as The Environment. Cell colors are as described in Fig. S3.

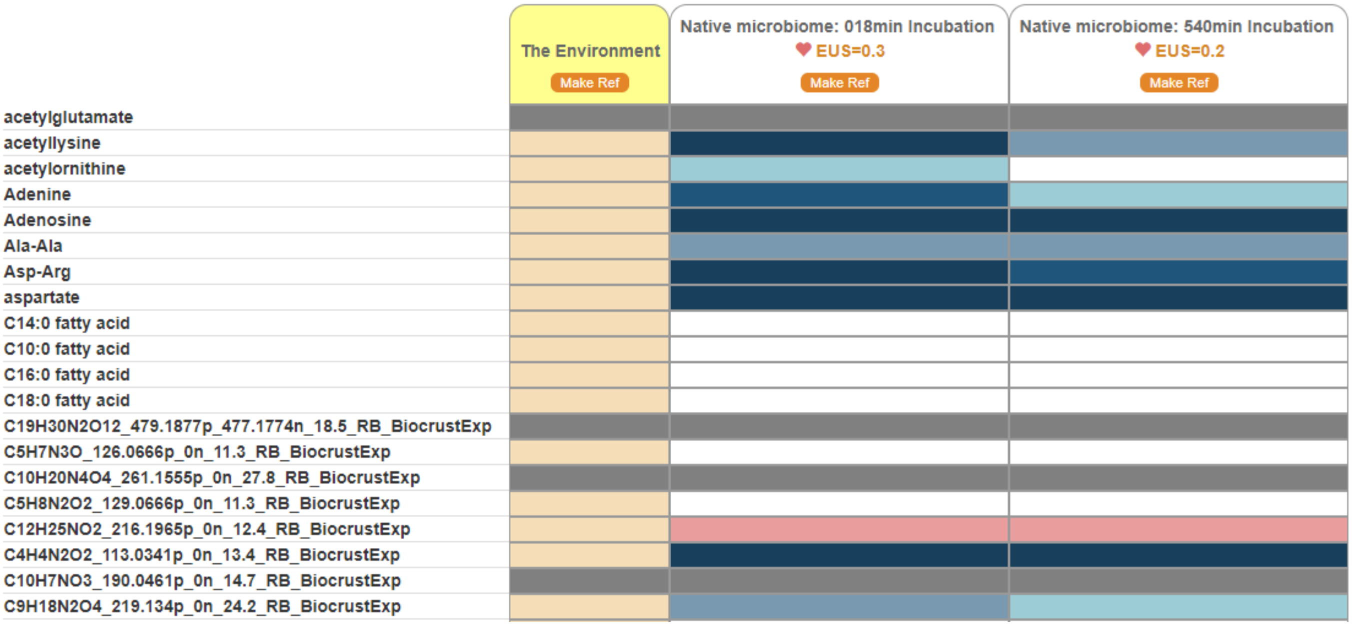

Figure S9. WoM One Environment view: native microbial communities. Metabolic changes in native communities activated within their native substrate may be entered using an un-activated sample as The Environment. In an experiment on Colorado biocrust, dormant samples were resuscitated by wetting with sterile water; porewater was extracted and analyzed with the initial wetting event used as the control ("The Environment") for comparison to the metabolite transformations resulting from microbial activities after incubation for 18 and 540 minutes [5]. Cell colors are as described in Fig. S3. Note: Only the first 20 metabolites from the table are included here.

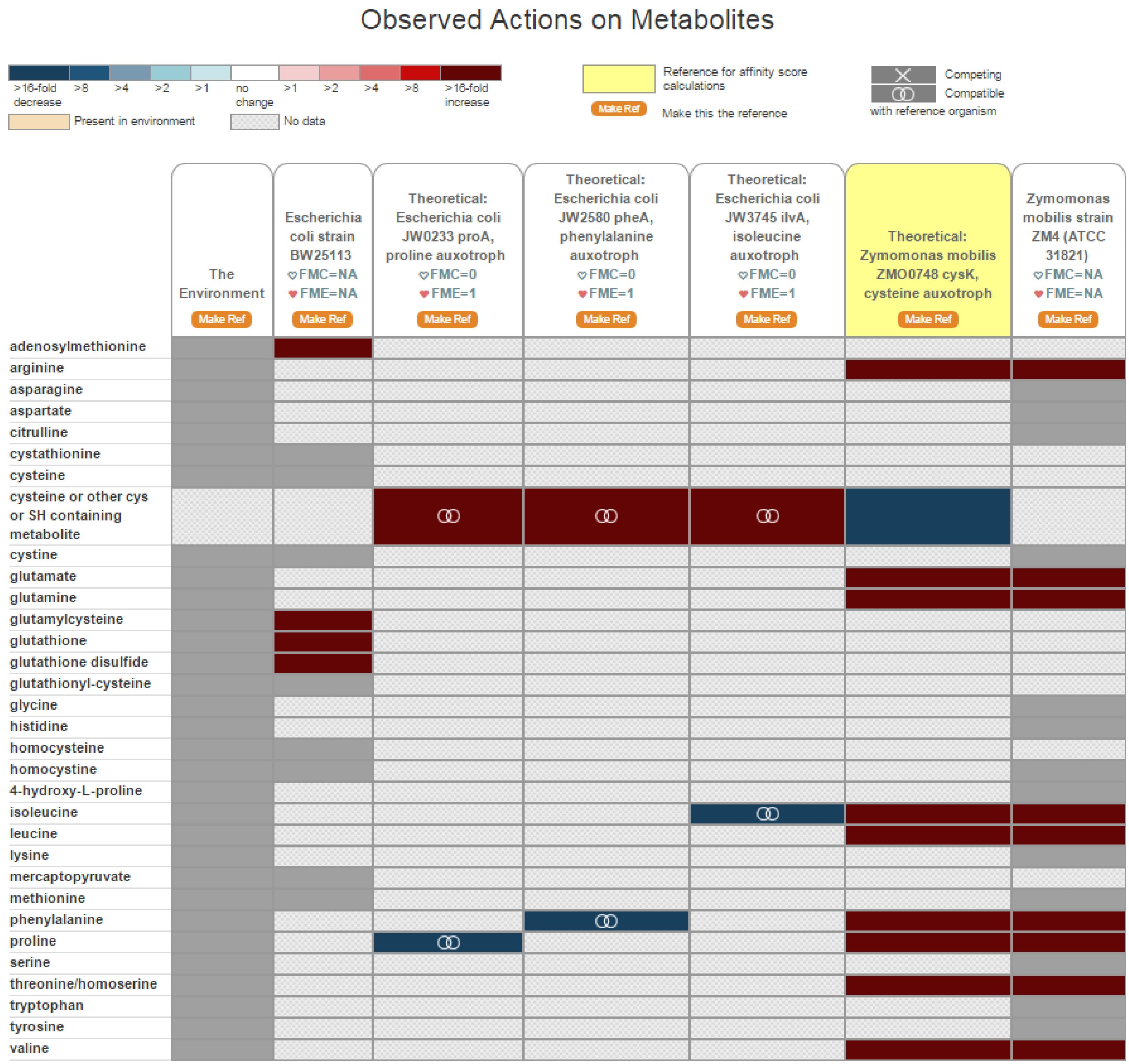

Figure S10. WoM One Environment, OCS-FME score: Predicted metabolite exchange potential between auxotrophs. *Zymomonas mobilis* minimal medium with glucose was analyzed before (control) and after (spent) mono-culture of wild type *E. coli* and *Z. mobilis* to assess synthesis and release of amino acid metabolites. Using this information, predictions were made for the rescue of auxotrophic mutants by metabolite exchange with the opposing strain in co-culture. Predictions were tested and validated via inability of isolate to grow in minimal medium, and growth of both strains in co-culture in minimal medium [7].

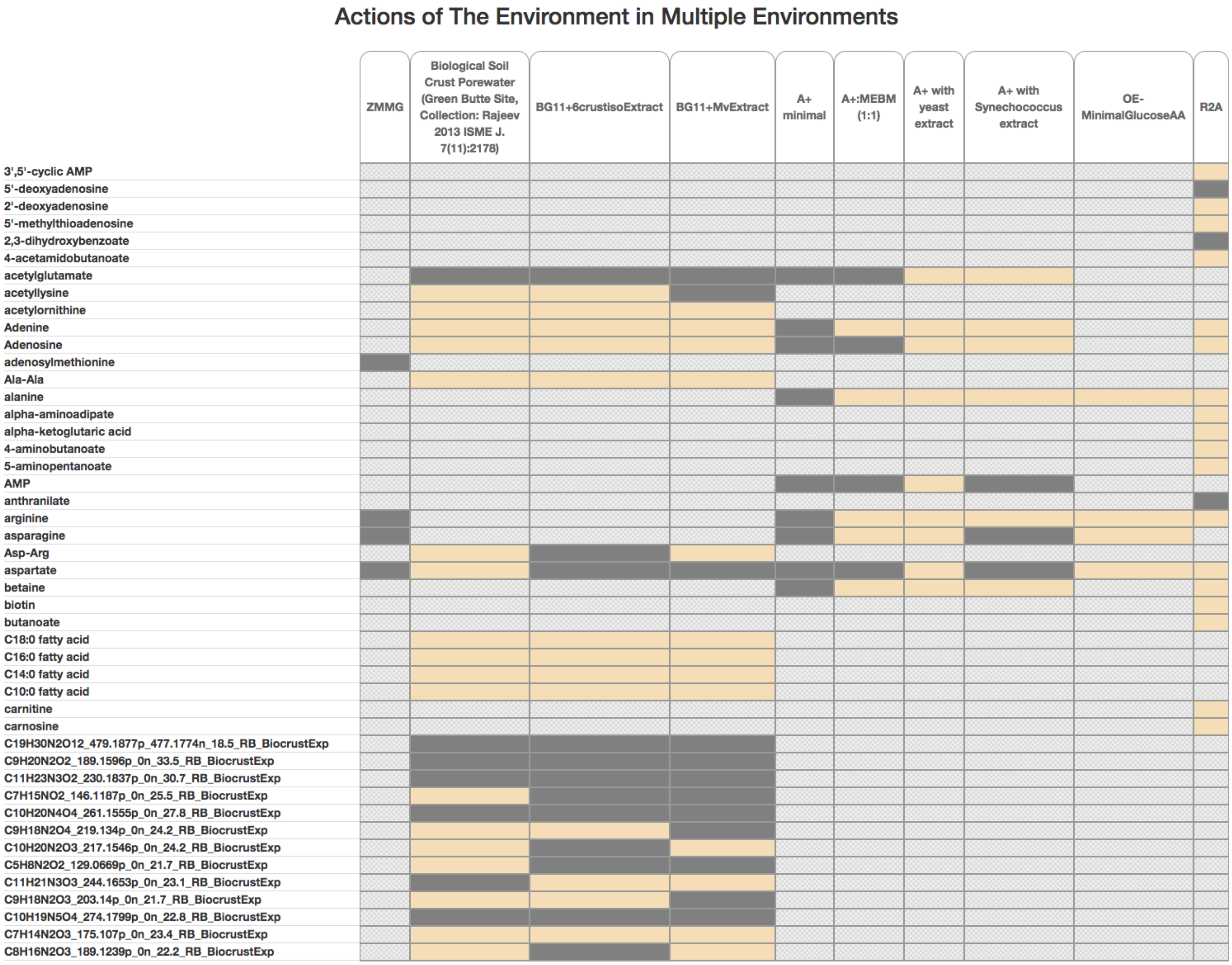

Figure S11. WoM One Organism view: Comparison of metabolite compositions of untransformed controls. The One Organism table view displays multiple types of untransformed control environments when "The Environment" is used to constrain the data. When viewed in this configuration, the only assertions visible will include: present (tan), not detected (solid gray) and no data available (checkered gray). Note that only the part of the metabolite table is shown here.

References

1. Watson DB, Kostka JE, Fields MW, Jardine PM. The oak ridge research center conceptual model. 2004;:1–55. https://public.ornl.gov/orifc/FRC-conceptual-model.pdf. Accessed 14 Jan 2014.
2. Bowen BP, Northen TR. Dealing with the unknown: metabolomics and metabolite atlases. J Am Soc Mass Spectrom. 2010;21:1471–6. doi:10.1016/j.jasms.2010.04.003.
3. Yao Y, Sun T, Wang T, Ruebel O, Northen T, Bowen B. Analysis of Metabolomics Datasets with High-Performance Computing and Metabolite Atlases. Metabolites. 2015;5:431–42. doi:10.3390/metabo5030431.
4. Baran R, Bowen BP, Northen TR. Untargeted metabolic footprinting reveals a surprising breadth of metabolite uptake and release by Synechococcus sp. PCC 7002. Mol Biosyst. 2011;7:3200–6. doi:10.1039/c1mb05196b.
5. Baran R, Brodie EL, Mayberry-Lewis J, Hummel E, Da Rocha UN, Chakraborty R, et al. Exometabolite niche partitioning among sympatric soil bacteria. Nat Commun. 2015;6:8289. doi:10.1038/ncomms9289.
6. Erbilgin O, Bowen BP, Kosina SM, Jenkins S, Lau RK, Northen TR. Dynamic substrate preferences predict metabolic properties of a simple microbial consortium. BMC Bioinformatics. 2017;18:57. doi:10.1186/s12859-017-1478-2.
7. Kosina SM, Danielewicz MA, Mohammed M, Ray J, Suh Y, Yilmaz S, et al. Exometabolomics Assisted Design and Validation of Synthetic Obligate Mutualism. ACS Synth Biol. 2016;5:569–76. doi:10.1021/acssynbio.5b00236.
