# Supplementary material for: Web of microbes (WoM): a curated microbial exometabolomics database for linking chemistry and microbes
Source: BMC Microbiol. 2018 Sep 12;18:115. doi: 10.1186/s12866-018-1256-y (PMC6134592; doi:10.1186/s12866-018-1256-y)
Supplement: Supplementary file 2 — Appendix A. Web of Microbes Terminology. (PDF 414 kb) [file 12866_2018_1256_MOESM2_ESM.pdf]

Web of Microbes (WoM): a curated microbial exometabolomics database for linking chemistry and microbes

Suzanne M. Kosina, Annette M. Greiner, Rebecca K. Lau, Stefan Jenkins, Richard Baran, Benjamin P. Bowen, and Trent R. Northen

Appendix A. Web of Microbes Terminology.

Views (Slice dimension)

The Web
Data is constrained by a selected environment; compounds are indicated by circles in the middle of the web connected by colored lines to organisms on the periphery with line colors indicating metabolite increase or decrease.

One Environment
Data is constrained by a selected environment. Data dimensions are presented in a 2 dimensional table with rows (metabolites) and columns (organisms). Table cell colors indicate metabolite assertions and symbols indicate interactions over a specified metabolite.

One Metabolite
Data is constrained by a selected metabolite.
Data dimensions are presented in a 2 dimensional table with rows (organisms) and columns (environments). Table cell colors indicate metabolite assertions and symbols indicate interactions over a specified metabolite.

One Organism
Data is constrained by a selected organism. Data dimensions are presented in a 2 dimensional table with rows (metabolites) and columns (environments). Table cell colors indicate metabolite assertions and symbols indicate interactions over a specified metabolite.

Assertions for microbial actions on metabolites

Decrease
The area under the curve of the extracted ion chromatogram of the metabolite of interest in the spent media is less than in the control media (t-test, $p<0.05$). “Decrease” indicates the removal of the detectable metabolite from the extracellular environment either by uptake to the periplasm/cytoplasm of the cell, extracellular degradation, extracellular modification, complexation/precipitation or changes in extraction solubility due to pH (or apparent pH in alcohol)

Increase
The area under the curve of the extracted ion chromatogram of the metabolite of interest in the spent media is greater than in the control media (t-test, $p<0.05$). “Increase” may indicate extracellular hydrolysis or depolymerization, excretion or cell lysis, enhanced extraction solubility due to pH (or apparent pH in alcohol)

No Change
The area under the curve of the extracted ion chromatogram of the metabolite of interest in the spend and control media are not statistically different from one another

No Data
The metabolite of interest was not searched for within the data for this particular metabolite-environment-organism combination.

Assertions for detection of metabolites in a control environment prior to microbial transformation

Not Detected
The metabolite was below the level of detection (determined experimentally for LCMS methods)

Present
The metabolite was above the level of detection in “The Environment” (control or preincubation media).

(Ex. the peak height for the extracted ion chromatogram is 1000 counts above the max height of the blank for the retention range of the compound of interest.

No Data
The metabolite of interest was not searched for within the data for this particular metabolite-environment combination.

Scores (available on One Environment slice only)

EUS
The Environmental Uptake Scores is the fraction of available metabolites in The Environment that are used (decreased) by an organism

OCS (Organismal Compatibility Scores):
OCS-FMC
The Fraction of Metabolites under Competition represents metabolites for which a scored organism may compete with a reference organism

OCS-FME
The Fraction of Metabolites for potential Exchange represents metabolites for which the reference organism has potential to ‘share’ with the scored organism

Data dimensions

Environment
A named pool of metabolites capable of sustaining growth of microorganisms and amenable to extraction and LCMS analysis procedures (ex. simple synthetic mixtures of metabolites, complex rich media, plant exudates, microbial lysates, soil extracts, etc).

Organism
The biotic agent(s) that transforms the environmental metabolite pool. This may be a bacterial, archaeal or eukaryotic isolate or a co-culture such as a synthetic mixture of isolates or a collection of organisms from an environmental sample. An untransformed control (“The Environment”) is included for comparison. As the transforming agent this identifier will include any incubation conditions (Ex. E. coli at 30°C vs E. coli at 25°C).

Metabolite
Any organic material of interest. This may be a class of compounds with similar structures (hexose isomers) or putative identification made by comparison to a reference standard or spectral database. Primarily, we are interested in metabolites that are produced and consumed by microorganisms and detectable by mass spectrometry.
